# Fermented fruit waste as a microbiome-active soil amendment: a review of field and greenhouse evidence (2009–2025)

**DOI:** 10.3389/fmicb.2026.1928844

**Published:** 2026-08-18

**Authors:** Nipapan Kanjana, Zhenni Zhao, Aning Fan, Ismail Shah, Muhammad Afaq Ahmed, Thiprada Poonsawat

**Affiliations:** 1 The National Key Engineering Lab of Crop Stress Resistance Breeding, School of Life Sciences, Anhui Agricultural University, Hefei, China; 2 Center for Crop Pest Detection and Control, Anhui Agricultural University, Hefei, China; 3 State Key Laboratory for Biology of Plant Diseases and Insect Pests, Institute of Plant Protection, Chinese Academy of Agricultural Sciences, Beijing, China; 4 Institute of Mountain Hazards and Environment, Chinese Academy of Sciences, Chengdu, China; 5 School of Forestry, University of Canterbury, Christchurch, New Zealand; 6 Proteins and Metabolites Team, Bioeconomy Science Institute, AgResearch Group, Lincoln, Canterbury, New Zealand; 7 Division of Innovative Botany, Department of Science and Bioinnovation, Faculty of Liberal Arts and Science, Kasetsart University Kamphaeng Saen Campus, Nakhon Pathom, Thailand

**Keywords:** circular agriculture, fermented fruit waste, nutrient cycling, rhizosphere ecology, soil health

## Abstract

Fruit residues generated across the global food supply chain constitute a rapidly growing, biologically reactive waste stream that, when left unmanaged, degrades environmental and agricultural quality, whereas controlled microbial fermentation offers a practical means of transforming this waste into stabilized, biologically active soil amendments. This review synthesizes evidence from field trials, greenhouse experiments, and microbiome studies published between 2009 and 2025 to evaluate the agronomic and microbiome-level effects of fermented fruit waste (FFW) as a soil amendment within circular waste-management frameworks. FFW application has been associated with increases in soil organic carbon of 12–24%, microbial biomass carbon of 38–64%, and plant biomass accumulation of 15–35% relative to unamended controls, with the magnitude of these effects varying substantially across studies according to feedstock composition, soil type, crop species, and fermentation method, rather than reflecting a single generalisable effect size. At the rhizosphere level, fermentation-derived substrates selectively recruit plant-growth-promoting rhizobacteria and phosphorus-solubilizing taxa, enhancing nutrient-use efficiency and microbial network resilience. These findings indicate that FFW functions not merely as a recycled nutrient input but as a biologically transformed amendment that actively restructures soil microbial communities and reinforces plant–soil feedback networks, while practical constraints such as phytotoxicity, salinity accumulation, pathogen survival, and feedstock contamination require evidence-based mitigation. However, standardized fermentation protocols, quantitative process-parameter thresholds, and long-term field validation remain limited, underscoring the need for future research integrating process optimization with multi-site field trials to enable reliable, microbiome-guided FFW application at scale.

## Graphical abstract

**Figure:**
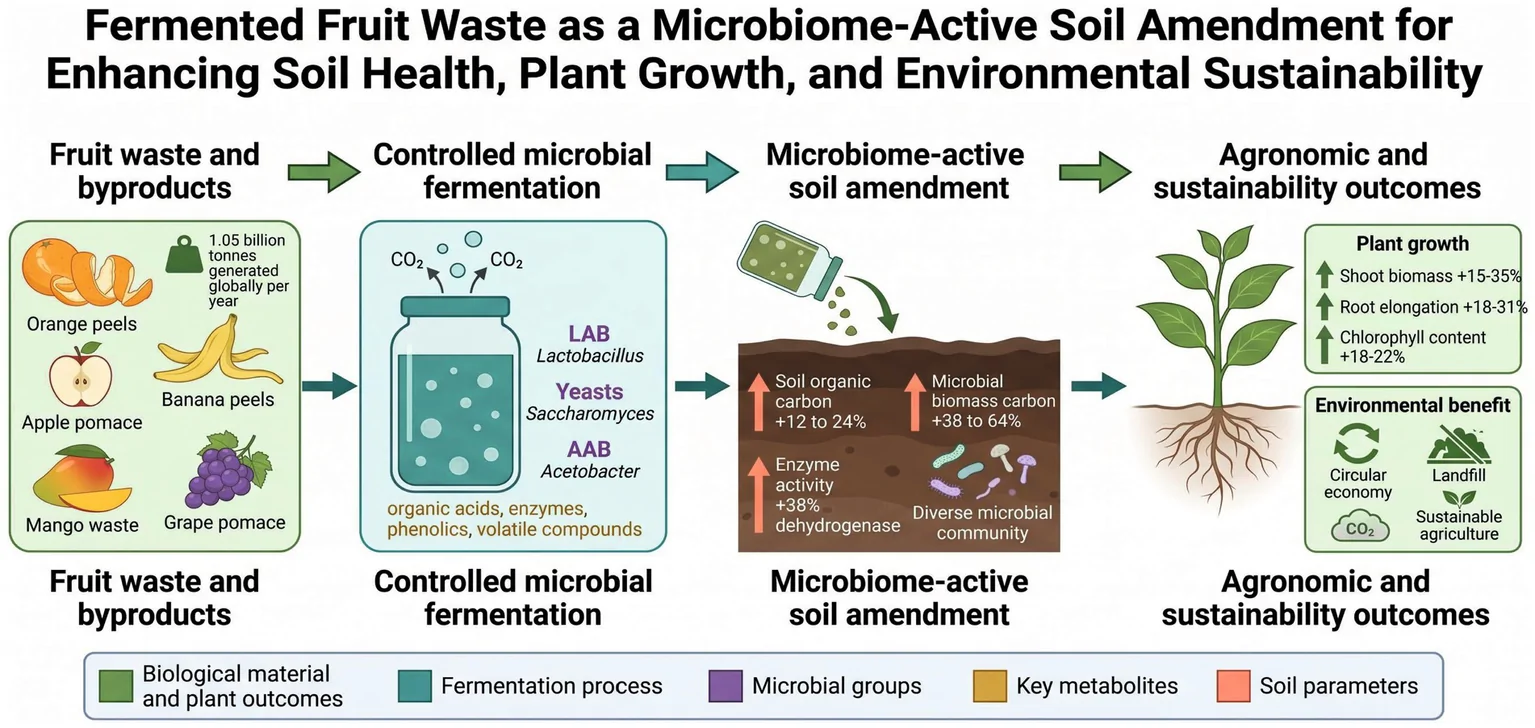

## Highlights

- Microbial fermentation converts unstable fruit residues into biologically active soil amendments.
- FFW reshapes soil microbial communities and selectively recruits rhizosphere beneficial taxa.
- Application increases soil organic carbon (12 to 24%) and plant biomass (15 to 35%) compared with unamended controls.
- FFW mitigates both abiotic and biotic plant stress through microbiome-mediated mechanisms.
- Standardized fermentation protocols and quality indicators are essential for safe field use.

## 1 Introduction

Fruit waste occupies a significant and growing fraction of the global food-waste burden. Across households, fresh-produce markets, restaurants, juice factories, and fruit-processing plants, fruit residues are generated on a daily and industrial scale, contributing to a food-waste stream that reached an estimated 1.05 billion tonnes globally in 2022 (United Nations Environment Programme, 2024). Unlike many agricultural by-products, fruit residues are biologically reactive rich in soluble sugars, organic acids, pectin, cellulose, hemicellulose, phenolic compounds, vitamins, and mineral nutrients including potassium, calcium, magnesium, and phosphorus (Sadh et al., 2018; Mahato et al., 2018). These properties make fruit waste both highly biodegradable and potentially valuable as an agricultural input, provided that appropriate biological transformation is applied before soil incorporation.

When left unmanaged, fruit residues decompose rapidly under natural conditions, producing odors, greenhouse gases (particularly methane under anaerobic landfill conditions), leachates, and nutrient losses that collectively burden waste-management infrastructure and degrade local environmental quality (Nanda and Berruti, 2021). This has driven growing interest in biological strategies capable of converting fruit waste from a liability into an agronomically useful resource. Among the available options, including composting, anaerobic digestion, biochar production, and bioactive compound extraction, microbial fermentation has attracted increasing scientific attention because of its capacity to transform labile fruit residues into chemically stabilized, biologically enriched, and agriculturally functional amendments at relatively low operational cost (Sarker et al., 2024; Mandal et al., 2024; Caldara et al., 2026). While certain agricultural residues, such as hemp, may contain allelochemical compounds that influence microorganisms and subsequently release allelochemical substances (Poonsawat et al., 2024; Kachel-Górecka et al., 2025).

Fermentation is fundamentally distinct from composting or simple decomposition. Under partially anaerobic or microaerophilic conditions, diverse microbial consortia including lactic acid bacteria (LAB), yeasts, acetic acid bacteria (AAB), Bacillus species, and filamentous fungi metabolize soluble carbohydrates, partially hydrolyse structural polymers, and produce a suite of organic acids, extracellular enzymes, antimicrobial compounds, and bioactive metabolites (Raman et al., 2022; Upadhyay et al., 2024). These transformations modify pH, nutrient solubility, amendment maturity, and the microbial composition of the fermented product, all of which influence how the amendment interacts with soil ecosystems after application (De Vuyst and Leroy, 2020; Pessione, 2012).

Applied specifically to fruit residues, this means fermented fruit waste retains living microbial cells and chemically active metabolites at the point of soil application, whereas composted fruit waste has typically undergone the same extended thermophilic decomposition process as other composted materials, resulting in a stabilized, humified product with a substantially different microbial and metabolite profile by the time of use. Because this review draws on several related but distinct fermentation concepts, four terms are clarified here and used consistently throughout. Soil fermentation describes the continued interaction between already-fermented material, such as FFW, and the soil environment following incorporation, rather than fermentation taking place within the soil itself (De Vuyst and Leroy, 2020). This is distinct from *in situ* fermentation, in which the fermentation process occurs directly within the soil or growing substrate rather than in a separate vessel beforehand (Raman et al., 2022). Most FFW preparation discussed in this review instead relies on solid-state fermentation (SSF), in which microbial activity proceeds on a solid or semi-solid substrate with low free water content, as opposed to submerged or liquid fermentation systems (Soon et al., 2025). Where fermentation or soil treatment involves the deliberate introduction of specific microbial strains or consortia to enhance a targeted function, such as nutrient solubilization or pathogen suppression, this is referred to as bioaugmentation, distinguishing it from the naturally occurring microbial succession that characterizes unamended fermentation (Gao et al., 2022).

The central scientific value of fermented fruit waste (FFW) lies not simply in its nutrient content, but in its biological activity. Soil health, defined as the sustained capacity of soil to function as a living system that supports plant growth, nutrient cycling, water regulation, and ecological resilience, depends critically on the activity and diversity of soil microbial communities (Lehmann and Kleber, 2015). Organic amendments can reshape these communities by modifying carbon availability, nutrient inputs, and substrate complexity, and FFW is particularly well suited to this role because its microbial metabolites, partially degraded carbon fractions, and preserved microbial cells collectively provide a suite of ecological inputs unavailable from conventional mineral fertilizers (Trivedi et al., 2020; Compant et al., 2019).

Despite the growing body of evidence supporting the agronomic value of FFW, several important knowledge gaps persist. Most prior reviews have treated fruit residues as a generic category of food waste, emphasizing composting or bioenergy applications over soil microbiome outcomes. Comparatively few studies have examined the distinct biological mechanisms through which fermented fruit residues reshape rhizosphere microbial ecology, regulate nutrient cycling, and modulate plant–soil feedback interactions.

This review synthesizes recent advances in soil fermentation with three aims: (1) to evaluate microbial and biochemical mechanisms that mediate soil responses; (2) to assess process parameters and scale-up strategies; and (3) to identify knowledge gaps and priority research needs. Drawing on peer-reviewed literature published primarily between 2009 to 2025, this narrative review evaluates FFW as a microbiome-guided soil amendment within circular waste valorisation frameworks, with specific relevance to waste management systems seeking biological alternatives to landfill disposal.

## 2 Microbial ecology and community dynamics

### 2.1 Microbiome-mediated mechanisms: from fermented fruit waste to rhizosphere function

The biological effects of FFW on plant–soil systems cannot be explained by physicochemical changes alone. A growing body of evidence demonstrates that organic amendments fundamentally reshape soil microbial community structure, metabolic function, and ecological network organization and these microbiome-level changes are recognized as the primary drivers of the agronomic benefits attributed to organic amendment application (Trivedi et al., 2020; Banerjee et al., 2019; Berendsen et al., 2012). FFW occupies a unique position in this landscape because it introduces not only transformed organic substrates but also living microbial cells, metabolites, extracellular enzymes, and signaling compounds that can actively recruit and shape rhizosphere communities following soil incorporation.

At the rhizosphere level, plant roots continuously release a diverse array of sugars, amino acids, organic acids, phenolics, flavonoids, and signaling molecules that selectively attract specific microbial taxa and establish the biochemical conditions of the immediate root environment (Bais et al., 2006). FFW disrupts and enriches this environment by introducing fermentation-derived labile carbon compounds and metabolites that alter microbial resource availability, niche structure, and competitive dynamics. Research on food-waste-derived fermented conditioners by Wang et al. (2024) demonstrated that long-term FFW application shifted soil bacterial community composition, enriching Proteobacteria, Actinobacteria, Acidobacteria, and Firmicutes, all phyla associated with nutrient transformation and plant growth promotion to dominant abundances of 65.95–77.52% of total bacterial sequences.

Among the functional groups most consistently enriched by FFW are plant-growth-promoting rhizobacteria (PGPR), phosphorus-solubilizing bacteria, potassium-mobilizing microorganisms, and nitrogen-transforming taxa (Compant et al., 2019; Pérez-Montaño et al., 2014). *Bacillus* species, *Pseudomonas* species, and Trichoderma species, all well-documented PGPR, have been associated with improved nutrient availability, phytohormone production, pathogen suppression, and rhizosphere stabilisation in soils amended with fermented organic materials. Lactic acid bacteria persisting from the fermentation process may also contribute to beneficial microbial consortia following soil application, through the production of organic acids, bacteriocins, and other antimicrobial compounds that reshape competitive dynamics among soil bacterial communities (Raman et al., 2022).

Disease suppression through microbial antagonism represents another key microbiome-mediated function of FFW. Beneficial rhizosphere microorganisms enriched by organic amendment application can inhibit soil-borne pathogens via nutrient competition, niche exclusion, antimicrobial metabolite production, hydrolytic enzyme secretion, and induction of systemic plant defence responses (Ongena and Jacques, 2008; Cha et al., 2016). Banerjee et al. (2019) demonstrated that organic amendment application increased the complexity and connectivity of soil microbial co-occurrence networks, a community property associated with greater ecological resistance to pathogen invasion and environmental perturbation. This suppressive soil effect is of particular relevance for sustainable agricultural systems seeking to reduce reliance on synthetic fungicides and bactericides.

Soil enzymatic activity within the rhizosphere is also strongly influenced by FFW application. Fermented amendments rich in microbial metabolites and easily degradable substrates stimulate extracellular enzyme production, including phosphatase, glucosidase, urease, cellulase, and dehydrogenase, within the root-influenced zone (Nannipieri et al., 2012; Zhang et al., 2024). These enzymes accelerate nutrient mineralization and organic matter turnover, increasing the availability of nitrogen, phosphorus, and potassium for root uptake. Zhang et al. (2024) reported significant increases in phosphatase and *β*-glucosidase activity in soils receiving fermented organic amendments, accompanied by substantial shifts in bacterial functional guilds associated with carbon cycling and nutrient transformation.

The agronomic effects of FFW are mediated by a specific chain of causation linking fermentation biochemistry to plant physiology, rather than by microbial community change in isolation. Organic acids, phenolic compounds, and antimicrobial metabolites generated during fermentation act first at the rhizosphere interface, where they are recognized by resident and introduced soil microorganisms and influence microbial community composition and function. This microbiome shift, in turn, alters the plant’s exposure to microbial signals and metabolic by-products, triggering physiological responses that extend beyond simple nutrient acquisition. For example, endophytic and rhizosphere bacteria capable of producing protease, cellulase, and siderophores have been shown to enhance host plant antioxidant enzyme activity, including superoxide dismutase and catalase, under abiotic stress conditions, directly linking microbial metabolic activity to plant stress physiology (Liang et al., 2024).

### 2.2 Effects of fermented fruit waste on soil physicochemical health

Soil physicochemical health underpins the productive capacity of agricultural ecosystems by governing nutrient availability, water retention, microbial activity, root penetration, and overall soil functional stability (Lehmann and Kleber, 2015). The application of FFW to agricultural soils modifies several of these properties simultaneously an outcome that distinguishes biologically transformed fruit residues from simple organic matter or mineral nutrient inputs. Quantitative evidence from recent field and greenhouse in Table 1 summarizes the chemical composition data from these trials.

**Table 1:** Key microbial groups involved in fruit waste fermentation, their optimal process conditions, and soil-relevant products.

Microbial group	Optimal pH	Temp (°C)	Key products	Soil benefit	References
Lactic acid bacteria	4.5–6.5	25–37	Lactic acid, enzymes	pH buffering, pathogen suppression	Raman et al. (2022)
*Saccharomyces* spp.	4.0–6.0	25–35	Ethanol, CO₂	Carbon input, microbial stimulation	De Vuyst and Leroy (2020)
*Acetobacter* spp.	5.0–6.5	25–30	Acetic acid	Nutrient mobilization	Pessione (2012)
*Bacillus* spp.	6.0–7.5	30–45	Enzymes, surfactins	PGPR enrichment, disease suppression	Upadhyay et al. (2024)
*Aspergillus* spp.	4.5–7.0	25–35	Cellulases, pectinases	Structural polymer degradation	Soon et al. (2025)

Soil organic matter (SOM) improvement is among the most consistently documented outcomes of FFW application. Fermented fruit residues contribute partially decomposed organic substrates, microbial biomass-derived carbon, extracellular metabolites, and humification precursors to the soil carbon pool. In a long-term field trial conducted over 6 years, Li et al. (2020) reported that annual application of a fermentation-derived food-waste conditioner increased soil organic matter accumulation by 5.3 g/kg alongside a fruit yield improvement of 556 kg/ha in strawberry production. Shorter-term studies by Kumar et al. (2025) recorded increases in soil organic carbon of 18–24% following a single season of fermented fruit-waste biofertiliser application at 5 t/ha in degraded Indian agricultural soils, a finding that should be weighted differently than Li et al.’s six-year result given the shorter observation window and lack of confirmed replication.

Soil aggregate stability and water retention are improved through fermentation-derived organic binding agents, microbial exopolysaccharides, and partially humified carbon fractions that facilitate macroaggregate formation (Six et al., 2004; Blanco-Canqui, 2017). These structural improvements are particularly relevant in sandy or compacted agricultural soils where organic matter depletion has compromised water-holding capacity and erosion resistance. Sarker et al. (2024) reported a 21% improvement in water-stable aggregate content following FFW application at 8 t/ha in a field trial with degraded tropical soil (exact replicate number not stated in the original publication), accompanied by a significant increase in soil water-retention capacity that persisted into the dry season.

The influence of FFW on soil pH and nutrient availability is closely tied to feedstock composition and fermentation maturity. Fruit-derived amendments containing substantial concentrations of organic acids and mineral cations, particularly potassium, calcium, and magnesium, can buffer soil pH through increased cation exchange processes while simultaneously facilitating mineral-bound phosphorus solubilization (Meena et al., 2014; Alburquerque et al., 2012). Multiple studies have documented enhanced phosphorus availability following amendment incorporation, with increases in Olsen-P of 15–30% reported in calcareous and alkaline soils (Kumar et al., 2025; Wang et al., 2023).

**Figure 1:**
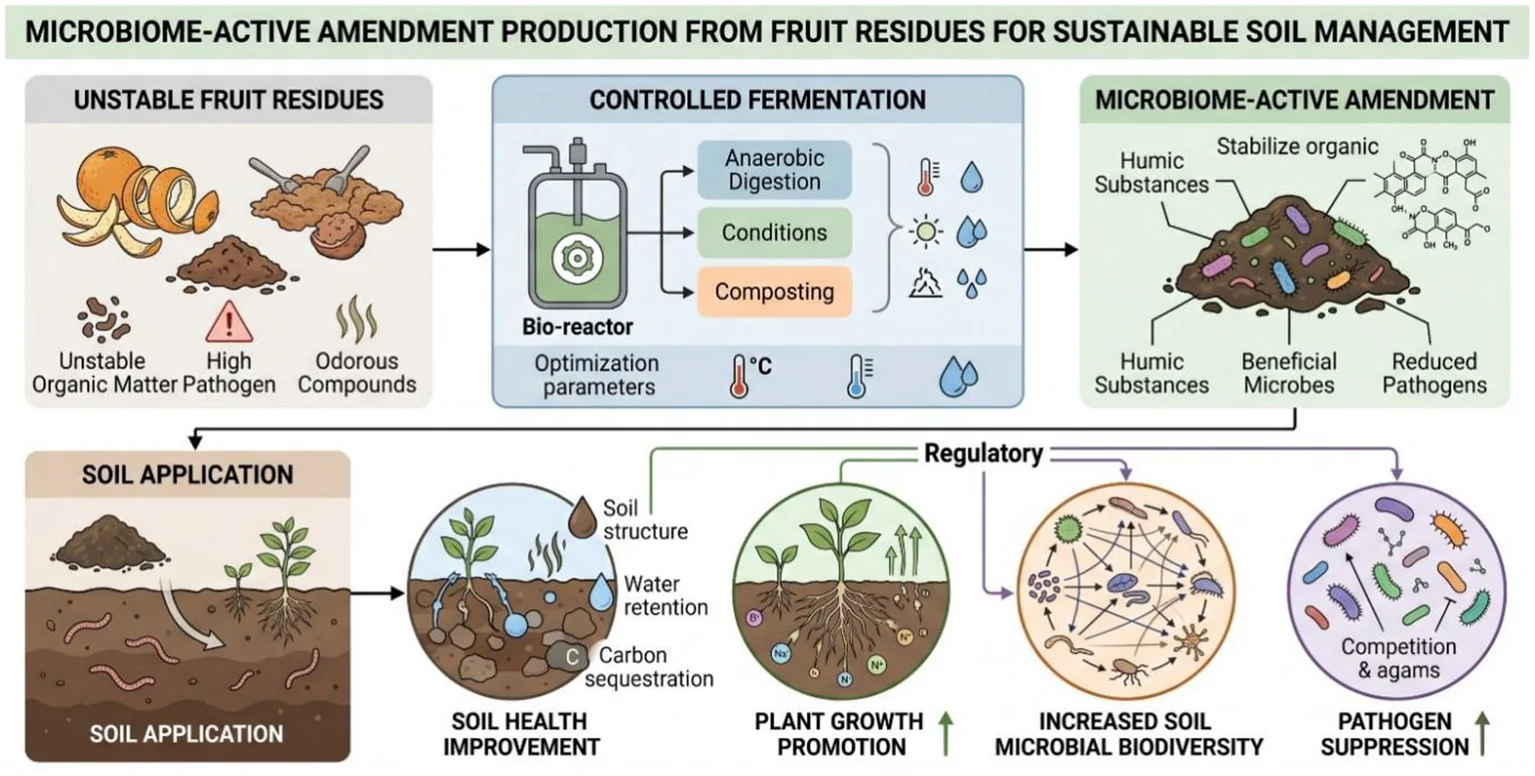
Fermentation as a biological transformation platform for fruit waste valorisation. Controlled microbial fermentation redirects unstable fruit residues through sequential biochemical transformation acidification, enzymatic hydrolysis, and metabolite enrichment into fermented fruit waste (FFW) as a microbiome-active soil amendment. Quality control assessment (pH, EC, C:N ratio, maturity index, phytotoxicity test) represents a critical checkpoint prior to agricultural application. FFW, fermented fruit waste; LAB, lactic acid bacteria; PGPR, plant growth-promoting rhizobacteria; VOCs, volatile organic compounds.

Soil enzyme activity is a particularly sensitive indicator of biological health and nutrient transformation efficiency. FFW amendments rich in microbial metabolites and degradable carbon substrates have been reported to stimulate the activity of dehydrogenase, urease, phosphatase, *β*-glucosidase, cellulase, and arylsulfatase within 4–8 weeks of soil incorporation in several studies (Nannipieri et al., 2012; Zhang et al., 2024), although the magnitude of this effect varies considerably by feedstock and soil type. For example, Soon et al. (2025) demonstrated a 38% increase in soil dehydrogenase activity in citrus-waste-fermented amendment trials, alongside a significant increase in microbial biomass carbon that was maintained for more than 12 weeks post-application. Controlled microbial fermentation redirects unstable fruit residues through sequential biochemical transformation encompassing acidification, enzymatic hydrolysis, and metabolite enrichment, into fermented fruit waste (FFW) as a microbiome-active soil amendment. Fermentation quality control, including assessment of pH, electrical conductivity (EC), carbon-to-nitrogen (C: N) ratio, maturity index, and phytotoxicity, represents a critical checkpoint prior to agricultural application. Properly stabilized FFW supports soil health improvement, microbiome restructuring, plant growth promotion, and circular agricultural sustainability. Poor fermentation quality resulting from inadequate process control poses significant risks of phytotoxicity and soil microbiome disruption. FFW, fermented fruit waste; LAB, lactic acid bacteria; PGPR, plant growth (Figure 1).

**Figure 2:**
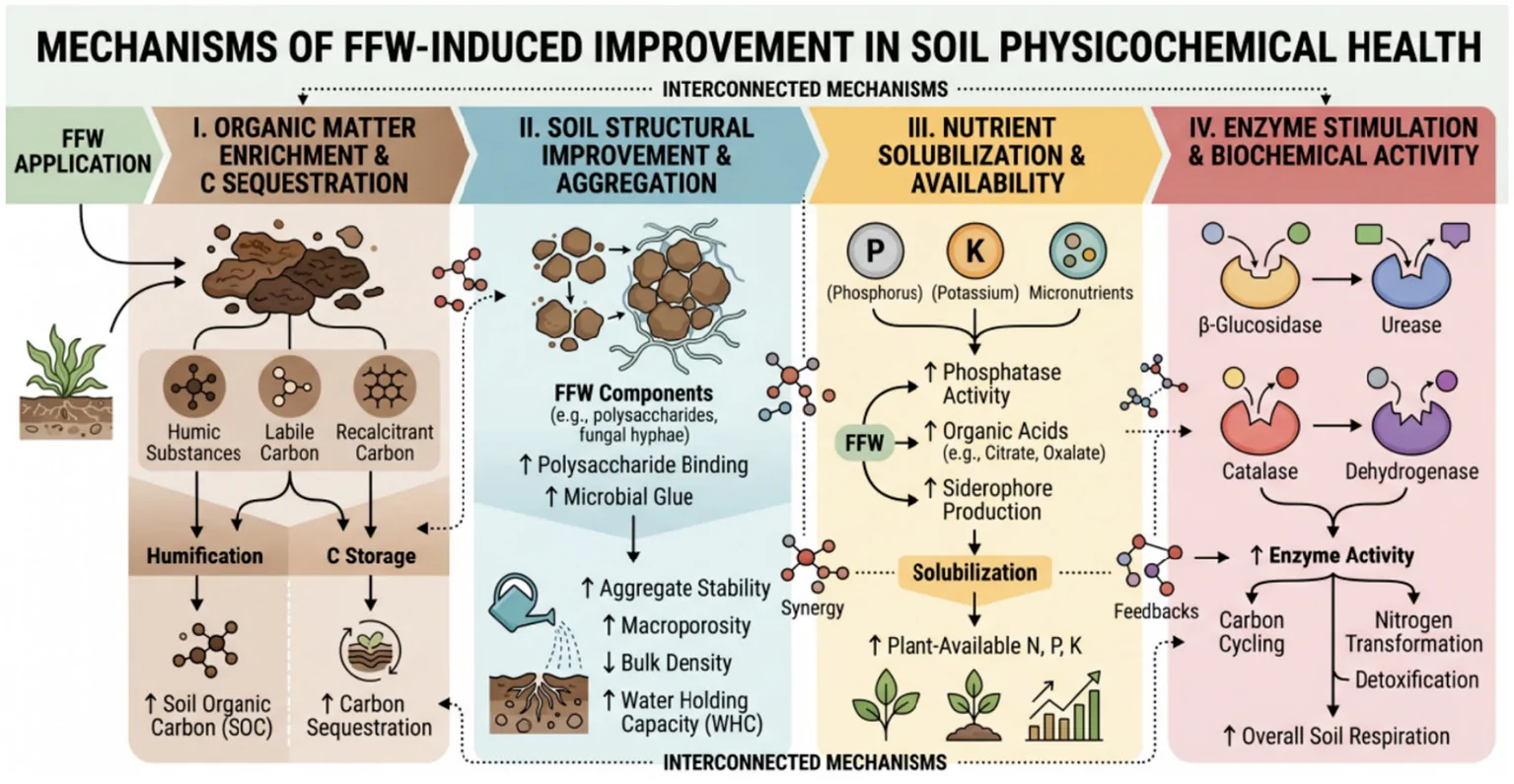
Effects of FFW on soil physicochemical health. FFW application influences soil functioning through five interconnected mechanisms: enhancement of soil organic matter and carbon dynamics; improvement of aggregate structure and water retention; regulation of pH and nutrient availability; stimulation of enzyme activity; and modulation of electrical conductivity. These modifications collectively improve ecosystem functionality and provide the environmental foundation for microbiome restructuring and sustainable plant performance. FFW, fermented fruit waste; SOC, soil organic carbon; CEC, cation exchange capacity; EC, electrical conductivity.

Despite these benefits, the physicochemical effects of FFW are context-dependent. Feedstock composition, fermentation method, amendment maturity, and soil type all influence outcomes substantially. Inconsistently processed amendments may generate products with excessive acidity, elevated electrical conductivity, or incomplete organic compound stabilisation conditions that can impair rather than improve soil functioning (Zaini et al., 2023; Bernal et al., 2009). Contaminant introduction from heterogeneous urban waste streams including pesticide residues, heavy metals, and microplastics represents an additional concern that necessitates careful feedstock selection and pre-screening (Sharma K. et al., 2017; Sharma B. et al., 2017; Corradini et al., 2021). The collective influence of FFW on soil physicochemical properties, spanning organic matter accumulation, aggregate stability, nutrient availability, enzyme activity, and Figure 2 summarizes electrical conductivity regulation, schematically, which illustrates the five interconnected mechanisms through which fermented amendments improve soil ecosystem functionality and establish the biological foundation for microbiome restructuring and plant growth promotion.

## 3 Biochemical pathways and metabolite production

### 3.1 Chemical diversity and bioactive constituents

The utility of fruit waste as a fermentation feedstock stems directly from its biochemical complexity. Unlike lignocellulosic crop residues, which are dominated by structurally recalcitrant cellulose and lignin, fruit-derived wastes contain an unusually high proportion of labile, biologically active compounds that are readily accessible to microbial metabolism (Lin et al., 2013; Sadh et al., 2018). This chemical richness makes fruit waste both highly fermentable and potentially valuable as a soil amendment, though it also introduces heterogeneity that must be managed carefully to ensure amendment consistency.

The soluble carbohydrate fraction principally glucose, fructose, and sucrose constitute the primary energy substrate for fermentative microorganisms and drives rapid pH reduction during early fermentation stages (Raman et al., 2022). Alongside these simple sugars, fruit residues contain structurally important polysaccharides including pectin, cellulose, and hemicellulose, which are degraded more slowly and contribute to longer-term soil organic matter accumulation following amendment application (Koul et al., 2022). Pectin-rich materials, such as citrus and apple peels, are of particular interest because pectinolytic microbial degradation generates soluble oligosaccharides and short-chain fatty acids that stimulate soil microbial activity well beyond the point of amendment incorporation (Awasthi et al., 2019).

Secondary metabolites represent a third biochemically important fraction. Phenolic acids, flavonoids, carotenoids, anthocyanins, tannins, and terpenoids are present across most fruit waste streams and retain partial biological activity even after processing or fermentation (Mahato et al., 2018; Ignat et al., 2011). These compounds can modulate rhizosphere microbial community structure by selectively stimulating or suppressing specific taxa, influencing extracellular enzyme activity, and participating in plant–microbe signaling interactions (Trivedi et al., 2020). Fermentation further transforms phenolic profiles; research by Faria et al. (2023) demonstrated that LAB fermentation of avocado leaf waste increased the total phenolic content by 55–71% depending on the bacterial strain, with corresponding increases in antioxidant activity that persisted in the fermented product.

**Figure 3:**
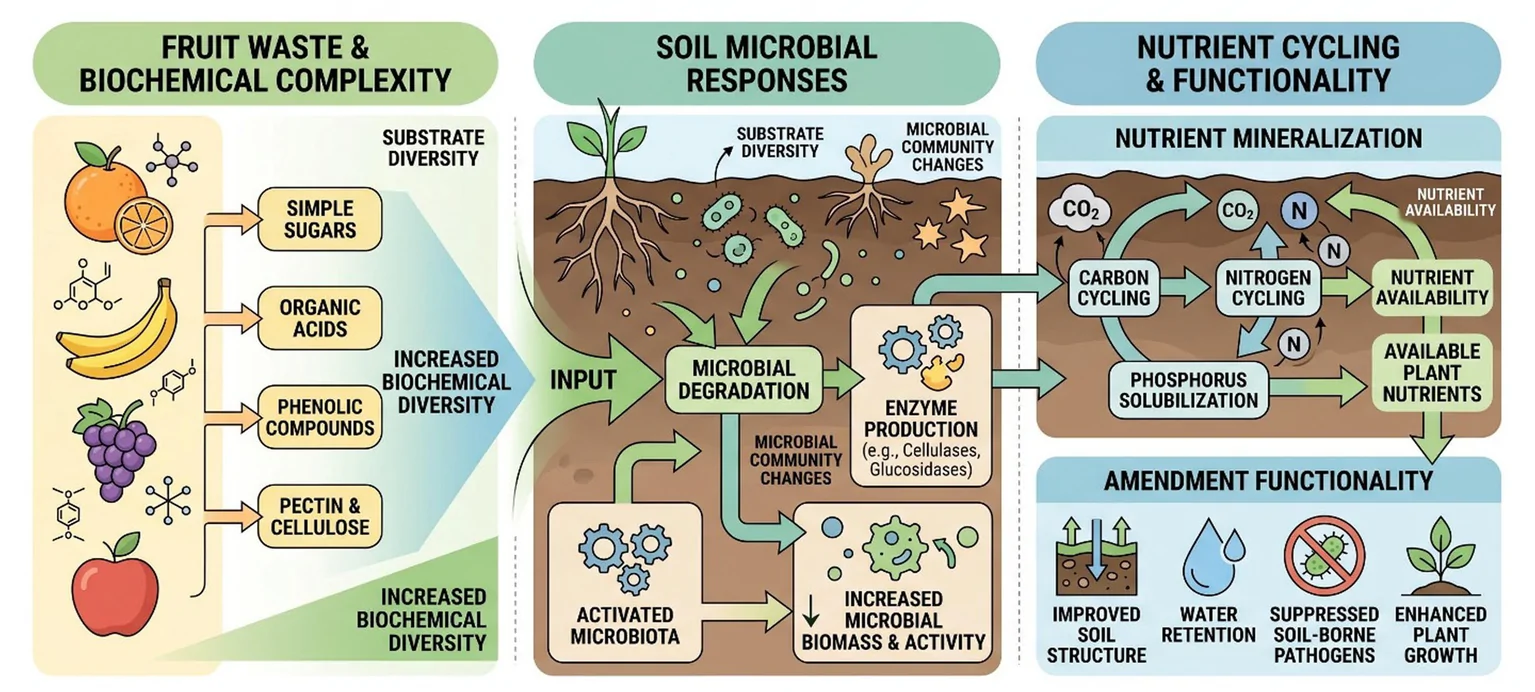
Biochemical constituents of fruit waste and their functional roles as soil amendments. Fruit-derived residues contain labile carbon substrates, structural polysaccharides, mineral nutrients, and bioactive secondary metabolites that collectively govern microbial stimulation, nutrient cycling, and rhizosphere functioning following soil incorporation. Phytotoxicity risk is associated with immature or improperly stabilized amendments applied at excessive rates.

Mineral nutrient composition varies substantially among fruit types and waste fractions (Table 2). Potassium is particularly abundant in banana, citrus, mango, and tropical fruit residues, whereas calcium-enriched fractions are associated with citrus peels and thick-rind fruits (Sharma K. et al., 2017; Sharma B. et al., 2017; Kaushik et al., 2025). These mineral profiles influence soil cation exchange capacity, osmotic balance, and plant nutritional status following amendment incorporation, and they should be considered when determining application rates for specific crop systems. Fruit-derived residues contain diverse chemical fractions including labile carbon substrates, mineral nutrients, structural polysaccharides, and bioactive secondary metabolites that collectively govern microbial stimulation, nutrient cycling, and rhizosphere functioning following soil incorporation. Phytotoxicity risk associated with immature or improperly stabilized amendments is also indicated (Figure 3; Table 3).

**Table 2:** Quantitative effects of fermented fruit waste and related organic amendments on soil physicochemical and biological properties.

Study	Feedstock	Application rate	SOC change	Microbial biomass change	References
Field trial, China	Food waste fermented	10 t/ha	+5.3 g/kg SOM	Proteobacteria ↑; Actinobacteria dominant	Li et al. (2020)
Greenhouse, India	Fruit waste fermented	5 t/ha	SOC + 18–24%	Microbial biomass C + 63.7%	Kumar et al. (2025)
Pot trial, Thailand	Citrus waste fermented	3% w/w	Organic matter +12%	Dehydrogenase +38%	Soon et al. (2025)
Field trial, Brazil	Mixed fruit fermented	8 t/ha	Aggregate stability +21%	Shannon diversity +0.43	Sarker et al. (2024)
Pot trial, China	Banana waste fermented	4 t/ha	Strawberry yield +556 kg/ha	Firmicutes and Actinobacteria enriched	Wang et al. (2024)

**Table 3:** Biochemical composition of major fruit waste types relevant to fermentation and soil amendment.

Fruit waste type	Moisture (%)	Major biochemical constituents	Key minerals	References
Citrus peel	72–85	Pectin (20–30%), limonene, flavonoids, organic acids	K, Ca, Mg	Mahato et al. (2018)
Banana peel	65–75	Starch, pectin, polyphenols, dopamine	K (3.6%), P, Mg	Sharma K. et al. (2017), Sharma B. et al. (2017)
Mango pomace	70–80	Cellulose, hemicellulose, polyphenols, mangiferin	Ca, Fe, K	Kaushik et al. (2025)
Apple pomace	75–85	Cellulose, pectin, polyphenols, malic acid	K, Ca	Sadh et al. (2018)
Grape pomace	55–65	Polyphenols, anthocyanins, tannins, tartaric acid	K, Fe, Mn	Lin et al. (2013)
Pineapple waste	80–88	Bromelain, citric acid, sugars, hemicellulose	Mn, K, Ca	Koul et al. (2022)

### 3.2 Physicochemical properties relevant to fermentation suitability

The physicochemical characteristics of fresh fruit waste directly determine fermentation efficiency and amendment quality. Fresh residues typically exhibit relatively low pH values (3.5–5.5) owing to the natural abundance of citric, malic, tartaric, and other organic acids (Sadh et al., 2018; De Vuyst and Leroy, 2020). During fermentation, these values decline further as LAB and yeast metabolism generates additional organic acids, accelerating substrate acidification and the suppression of spoilage-associated microorganisms (Raman et al., 2022). This self-acidification is agronomically beneficial in pathogen-management terms, but excessively acidic final products, particularly those with pH below 3.5, may inhibit seed germination and root elongation if applied without adequate stabilisation or pH adjustment (Bernal et al., 2009).

High moisture content (65–88%) is a universal characteristic of fruit residues and a critical determinant of fermentation dynamics (Sadh et al., 2018). While adequate moisture supports microbial activity, excess water reduces oxygen diffusion in aerobic fermentation systems and promotes the formation of undesirable fermentation by-products. Pre-treatment steps such as partial drying, size reduction, and moisture adjustment are therefore important preparation stages that influence the reproducibility of FFW quality across production batches (Zaini et al., 2023).

**Figure 4:**
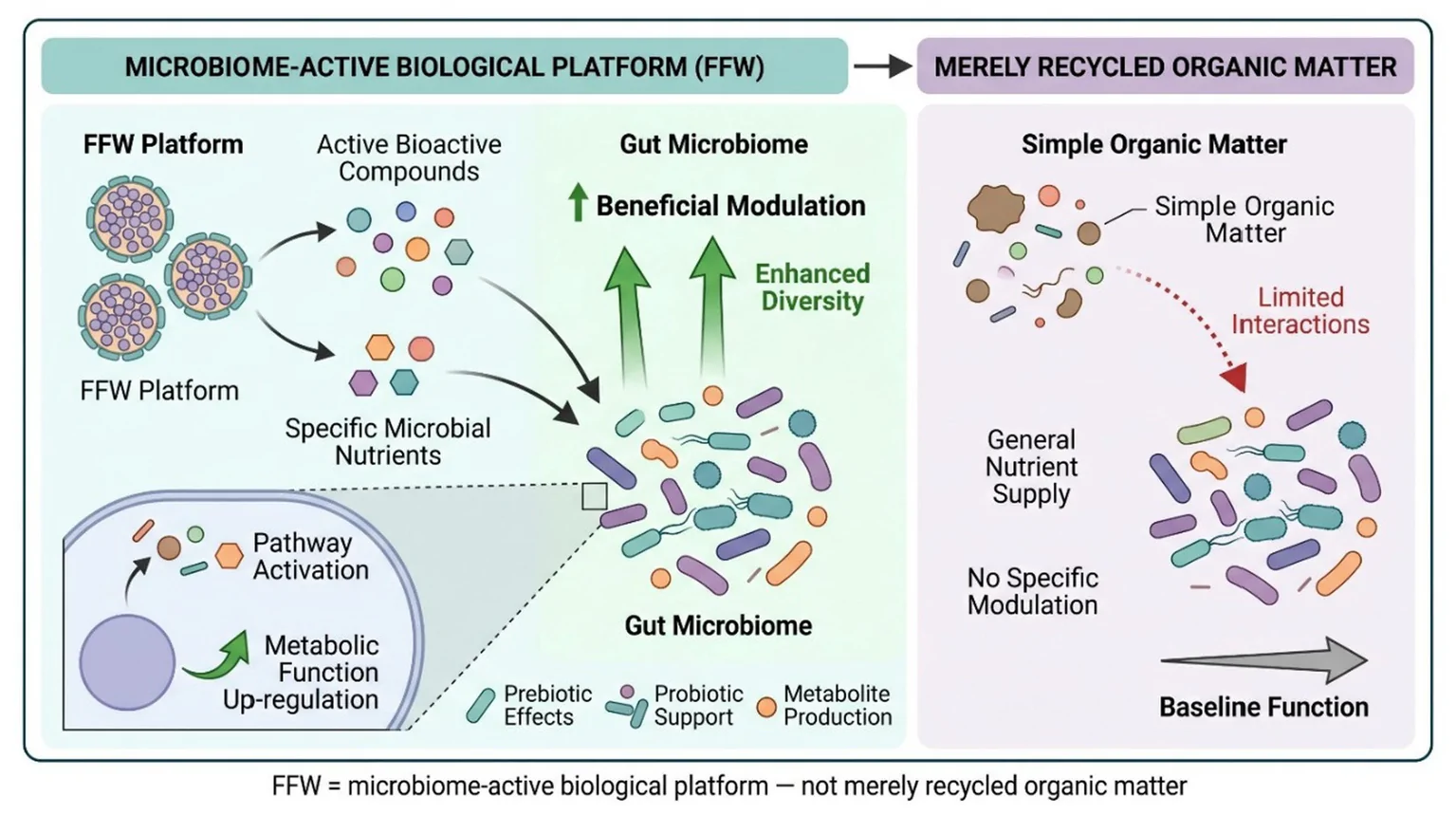
Fermented fruit waste (FFW) as a microbiome-active biological platform. FFW delivers specific bioactive compounds and microbial nutrients that drive beneficial modulation and enhanced diversity of the soil microbiome, with intracellular pathway activation and metabolic function upregulation. In contrast, conventional organic matter provides general nutrient supply with limited microbial interactions. FFW, fermented fruit waste.

Perhaps the most important physicochemical criterion for soil application is amendment maturity the degree to which labile organic compounds have been sufficiently transformed to prevent phytotoxic effects after soil incorporation. Maturity is typically assessed through a combination of indicators including pH, electrical conductivity, C: N ratio, germination index, and volatile organic acid profile (Bernal et al., 2009). A germination index exceeding 50% is the most commonly used biological threshold for safe soil application, though more comprehensive microbiome-based quality indicators are increasingly being proposed as supplements to physicochemical screening (Trivedi et al., 2020). FFW delivers active bioactive compounds and specific microbial nutrients that drive beneficial modulation and enhanced diversity of the soil microbiome, accompanied by intracellular pathway activation and metabolic function upregulation. In contrast, conventional organic matter provides only general nutrient supply with limited microbial interactions, maintaining baseline microbiome function without specific modulation. We define a microbiome-active amendment as one that meets three criteria that conventional organic amendments typically do not: first, it introduces living, functionally selected microbial consortia (e.g., LAB, yeasts, *Bacillus* species) rather than a microbial population shaped primarily by extended environmental succession (Liu et al., 2024); second, it retains bioactive fermentation metabolites, such as organic acids and antimicrobial compounds, that remain chemically active at the point of application rather than being progressively degraded and humified during a prolonged maturation period (Bernal et al., 2009); and third, it acts primarily through targeted recruitment and competitive exclusion of specific microbial functional groups, rather than through passive nutrient release alone. Compost and manure, by comparison, typically undergo extended aerobic maturation spanning weeks to months, during which microbial community composition shifts through successive mesophilic, thermophilic, and curing phases driven by changing temperature and substrate availability (Liu et al., 2024), resulting in a comparatively diffuse and less targeted influence on the receiving soil microbiome by the time of application. FFW, fermented fruit waste (Figure 4).

## 4 Process parameters and optimization

Controlled microbial fermentation transforms fruit waste from an unstable, rapidly decomposing organic residue into a chemically enriched and biologically functional amendment. This transformation is not a passive decomposition event; it is an ecologically structured process in which microbial succession, substrate competition, and metabolic cross-feeding collectively determine the chemical composition, biological activity, and agricultural suitability of the final product (De Vuyst and Leroy, 2020; Sarker et al., 2024).

The fermentation process typically begins with the rapid proliferation of facultative anaerobes primarily yeasts and heterofermentative LAB, which consume soluble sugars and generate lactic acid, ethanol, and carbon dioxide within the first 24–72 h (Raman et al., 2022). This early phase drives acidification that selectively suppresses spoilage-associated microorganisms and establishes the pH conditions that favor subsequent fermentation stages. As the fermentation matures, Bacillus species, pectinolytic fungi, and AAB assume increasing ecological importance, producing extracellular enzymes including cellulases, pectinases, proteases, and xylanases that depolymerise structural plant materials and release smaller bioavailable compounds (Upadhyay et al., 2024; Soon et al., 2025).

Metabolomic investigations by Faria et al. (2023) and Zhang et al. (2024) have demonstrated that microbial fermentation substantially restructures the chemical profile of fruit-derived substrates, altering the abundance of phenolic acids, flavonoids, amino acids, volatile organic compounds, and antioxidant metabolites through biotransformation pathways that are both strain-specific and substrate-dependent. These metabolic shifts are not trivial from an agronomic perspective: low-molecular-weight metabolites generated during fermentation can participate directly in rhizosphere signaling, microbial community selection, and plant stress responses after soil application (Trivedi et al., 2020). An overview of key fermentation parameters and Table 1 presents the fermentation process parameters and their associated microbial groups.

To critically evaluate the evidence base rather than simply list findings, key studies on fermented and composted organic waste as a soil amendment are compared in Table 4, spanning feedstock type, fermentation or composting method, experimental scale, and reported outcomes. Across these studies, organically treated soils consistently show higher soil organic carbon, nutrient availability, or plant growth relative to unamended controls, indicating that this directional effect is robust across feedstocks and geographic contexts. However, the comparison also reveals an important limitation: only two of the eight studies test fruit waste as a specifically named feedstock, while the remainder rely on the closest verifiable analogs (fermented bread, bokashi, olive mill waste, fungal-fermented straw, and compost from municipal solid waste digestate or manure). Several of these studies also use aerobic composting rather than the LAB/yeast/*Bacillus*-driven fermentation this review otherwise focuses on, a mechanistic distinction summarized in the Methodological Limitations column of Table 5.

**Table 4:** Risk categories, causative factors, potential impacts, and mitigation strategies for FFW soil amendments.

Risk category	Causative factor	Potential impact	Mitigation strategy	References
Phytotoxicity	Organic acid accumulation, ethanol	Seed germination inhibition, root damage	Maturity index >50 (germination test)	Bernal et al. (2009)
Salinity stress	Soluble salt accumulation	Osmotic stress, EC > 4 dS/m toxic	EC monitoring; dilution before application	Rengasamy (2010)
Pathogen survival	Incomplete fermentation	Foodborne pathogen introduction	pH < 4 maintained; thermal validation	Raman et al. (2022)
Pesticide residues	Contaminated feedstock	Soil and crop contamination	Feedstock screening; certified organic sources	Corradini et al. (2021)
Antibiotic resistance	Urban waste stream	ARG transfer to soil microbiome	Metagenomics screening; feedstock selection	Wang et al. (2023)
Nutrient imbalance	High C: N ratio feedstock	N immobilization; crop N deficiency	C: N adjustment <25:1 before application	Bernal et al. (2009), Breza and Grandy (2025)

**Table 5:** Summary of key studies on fermented and composted organic waste as a soil amendment, including feedstock, process, experimental scale, key outcomes, and methodological limitations.

Feedstock/Soil type	Fermentation method	Scale	Key outcomes	Methodological limitations	Study (Author, Year)
Wasted bread; alkaline soil (Italy)	Amylase treatment + LAB fermentation	Pot trial, 1 cycle	SOC + 37%, N + 40%; leaf count up to 1.7x control; no phytotoxicity	1 season; pot-scale; replicate n unstated	Cacace et al. (2022)
Mixed bokashi ingredients; potting mix (USA)	Bokashi fermentation, 12-day maturation	Seedling trial	Higher chlorophyll and biomass in all bokashi types; more beneficial microbes	Preprint, not peer-reviewed; seedling stage only	Abo-Sido et al. (2021)
Olive mill waste; olive grove (Italy)	Composting (aerobic)	Long-term field trial	Higher C sequestration in soil, prunings, and fruit	Composting, not LAB/yeast fermentation; 1 crop system	Regni et al. (2017)
Corn straw; greenhouse pots (China)	Solid-state fungal fermentation, 30 days	1-season greenhouse	Best rate: tomato yield +30%, lycopene/vitamin C + 20–40%	1 season; not fruit waste; in press	Song et al. (2026)
Urban waste incl. Fruit waste (P3); field (Indonesia)	Fermentation to liquid fertilizer	RBD field trial	All treatments raised eggplant/Bok Choy yield; fish/blood waste beat fruit waste	Fruit waste 1 of 6 feedstocks; 1 cycle	Haryanta et al. (2023)
Mango, banana, jackfruit peel; soil (Bangladesh)	Composting, 6-month maturation	Factorial CRD, 3 reps	Mango: highest N, P; banana: highest K; jackfruit: highest organic C	Composting, not fermentation; no yield outcome measured	Paul et al. (2024)
Compost/biochar/lime; pomelo orchard (Vietnam)	Aerobic composting (comparator)	3-year field trial	Compost/biochar: 1.5x yield vs. chemical fertilizer; higher profit	Not fermented; feedstock not fruit-specific	Van Dang et al. (2022)
MSW digestate compost vs. manure; apple orchards (Italy)	Aerobic composting vs. manure maturation	3-year, 3 orchards	Compost raised N and SOM over time; manure raised P and K	Not fermented; feedstock not fruit waste; site-dependent	Bona et al. (2022)

**Figure 5:**
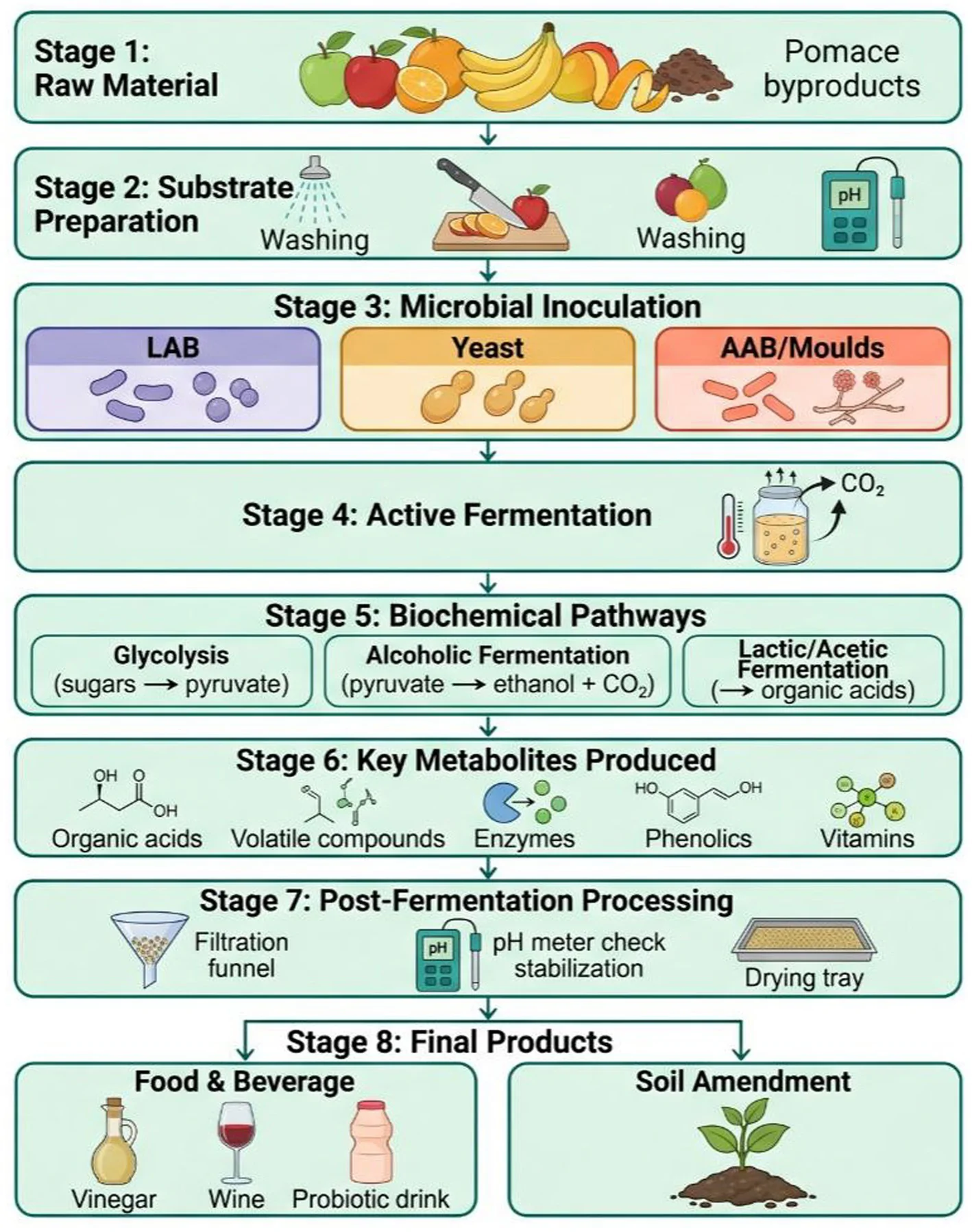
Schematic of the organic fruit fermentation pathway. Raw fruit and fruit waste (peels, pulp, pomace) undergo substrate preparation before inoculation with lactic acid bacteria (LAB), yeasts, and acetic acid bacteria (AAB) or moulds. Active fermentation proceeds through glycolysis, alcoholic fermentation, and lactic/acetic fermentation, generating key bioactive metabolites. Post-fermentation processing yields two principal product streams: food and beverage applications and soil amendments for plant growth promotion. LAB, lactic acid bacteria; AAB, acetic acid bacteria; CO₂, carbon dioxide.

Fermentation quality is strongly governed by process parameters, including temperature, oxygen availability, inoculum composition, carbon-to-nitrogen ratio, moisture level, pH, and fermentation duration all influence the microbial succession patterns and the biochemical profile of the final product (De Vuyst and Leroy, 2020). Poorly controlled fermentation can lead to accumulation of volatile fatty acids, excess ethanol, or unstable intermediate metabolites that impair seed germination and disrupt rhizosphere microbial equilibrium after soil application (Bernal et al., 2009; Alburquerque et al., 2012). This variability underscores the critical importance of establishing standardized fermentation protocols and rigorous maturity assessment frameworks before FFW is deployed at field scale. Raw fruit and fruit waste (peels, pulp, pomace) undergo substrate preparation before inoculation with lactic acid bacteria (LAB), yeasts, and acetic acid bacteria (AAB) or moulds. Active fermentation proceeds through glycolysis, alcoholic fermentation, and lactic/acetic fermentation, generating key bioactive metabolites including organic acids, volatile compounds, enzymes, phenolics, and vitamins. Post-fermentation processing yields two principal products: food and beverage applications and soil amendments for plant growth promotion. LAB, lactic acid bacteria; AAB, acetic acid bacteria; CO₂, carbon dioxide (Figure 5).

## 5 Scale-up, engineering, and techno-economic/environmental assessment

Laboratory and greenhouse studies leave little doubt that FFW alters soil chemistry and microbial community structure in agronomically favorable ways. Whether these benefits survive the transition to field-deployable, economically defensible production systems, however, remains far less settled. This gap separates FFW from more mature branches of the solid-state fermentation (SSF) literature, where engineering feasibility and process economics have received sustained attention.

Bioreactor design is the first obstacle. Heat and mass transfer, trivial to manage in a Petri dish or small flask, become genuine engineering problems once substrate volumes scale beyond bench level; uneven aeration or temperature gradients within a larger reactor can undermine the very microbial succession patterns that make fermentation effective in the first place (Cano-González et al., 2026). Tray, rotating-bed, and packed-bed configurations each offer partial solutions, but no study to date has systematically compared these designs for fruit-waste substrates specifically, leaving practitioners to extrapolate from adjacent SSF applications rather than fruit-waste-specific engineering data (Cano-González et al., 2026). Downstream handling, dewatering, stabilisation, packaging, is easy to overlook on paper and expensive to ignore in practice, often accounting for a disproportionate share of both cost and environmental burden (Cano-González et al., 2026).

Economic modeling of related organic-waste fermentation systems offers a useful, if indirect, benchmark. Muhammad and Rosentrater (2020) simulated a commercial-scale food-waste fermentation plant and found that fermentation residues retained resale value as a soil-amendment co-product, meaningfully improving the economics of the overall process rather than existing merely as a disposal cost (Okoro et al., 2019). Scale, in other words, is not a straightforwardly favorable variable, and FFW production planning would do well to treat it as an optimization problem rather than a target to maximize.

Life-cycle assessment (LCA) tells a similarly mixed story (Orlandella and Fiore, 2025), reviewing 98 LCA studies of agricultural-waste-derived biofertilizers, found that global warming potential, eutrophication, and acidification impacts varied considerably with feedstock and functional unit, and that full-scale composting and anaerobic digestion often carried a net-positive emissions burden despite their landfill-diversion benefits. Perhaps more telling for the present review, fewer than a third of the studies they examined paired environmental assessment with any economic analysis at all. This is not a minor omission: without joint LCA-TEA evaluation, it is difficult to identify which amendment strategies are genuinely both sustainable and commercially viable, as opposed to merely one or the other.

The barriers to adoption, finally, are not solely technical (Makepa and Chihobo, 2024) point to capital cost, regulatory ambiguity, and weak market competitiveness as recurring, mutually reinforcing obstacles across the broader biorefinery sector, while (Ray et al., 2025) note that techno-economic and lifecycle data specific to fruit and vegetable waste-derived SSF products have simply not kept pace with the rate of laboratory innovation in this space. The result is an evidence base that is lopsided: rich in agronomic promise, thin in the process engineering and economic scaffolding needed to act on it.

Closing this gap will require three things that the FFW literature has not yet produced: bioreactor engineering research conducted on fruit-waste substrates rather than borrowed from adjacent SSF applications; scenario-based techno-economic modeling sensitive to regional feedstock costs and infrastructure; and integrated LCA-TEA frameworks that assess environmental and economic performance together rather than as separate, disconnected exercises. Until these exist, the case for FFW at field scale will remain persuasive in principle and largely untested in practice.

## 6 Applications and limitations

### 6.1 Mechanisms of plant growth regulation

Plant responses to FFW application are mediated through three interconnected pathways: direct physicochemical improvement of the soil growth environment, microbiome-mediated nutrient transformation and phytohormone delivery, and metabolic-physiological regulation through fermentation-derived bioactive compounds (Trivedi et al., 2020; Compant et al., 2019). These pathways operate simultaneously and reinforce one another, producing growth responses that exceed those achievable through nutrient supplementation alone.

The most consistently documented agronomic outcome is stimulation of plant biomass accumulation. In pot and field trials across multiple crop species including tomato, wheat, strawberry, lettuce, maize, and rice, FFW application at rates of 3–10 t/ha or 2–6% soil incorporation produced shoot biomass increases of 15–35%, root elongation improvements of 18–31%, and chlorophyll content increases of 18–22% compared with unamended controls (Kumar et al., 2025; Zaini et al., 2023; Soon et al., 2025). These growth responses are summarized in Table 6.

**Table 6:** Reported effects of fermented fruit waste and related amendments on plant growth parameters across multiple crop species.

Crop	FFW type	Application	Growth parameter	Outcome	References
Tomato	Food waste fermented	5 t/ha	Shoot biomass	↑ 28–35% vs. control	Kumar et al. (2025)
Wheat	Fruit pomace fermented	3 t/ha	Chlorophyll content	↑ 22% (SPAD value)	Zaini et al. (2023)
Strawberry	Food waste conditioner	6-yr application	Fruit yield	+556 kg/ha	Li et al. (2020)
Lettuce	Citrus waste fermented	2% soil incorporation	Root length	↑ 18–31%	Soon et al. (2025)
Maize	Mixed fruit fermented	4 t/ha	Grain yield	↑ 15–19%	Mandal et al. (2024)
Rice	Banana waste fermented	5 t/ha	N uptake efficiency	↑ 24%	Sarker et al. (2024)

Root system architecture is particularly responsive to FFW application. Enhanced root branching, root hair development, and elongation are partly attributable to PGPR enrichment within the rhizosphere, as taxa producing indole-3-acetic acid (IAA), gibberellins, cytokinins, and siderophores directly regulate root development and nutrient uptake capacity (Pérez-Montaño et al., 2014). In parallel, improved soil aggregate stability and water retention following FFW incorporation creates a more physically favorable rooting environment that reduces mechanical impedance and supports rhizosphere microbial colonization (Blanco-Canqui, 2017).

**Figure 6:**
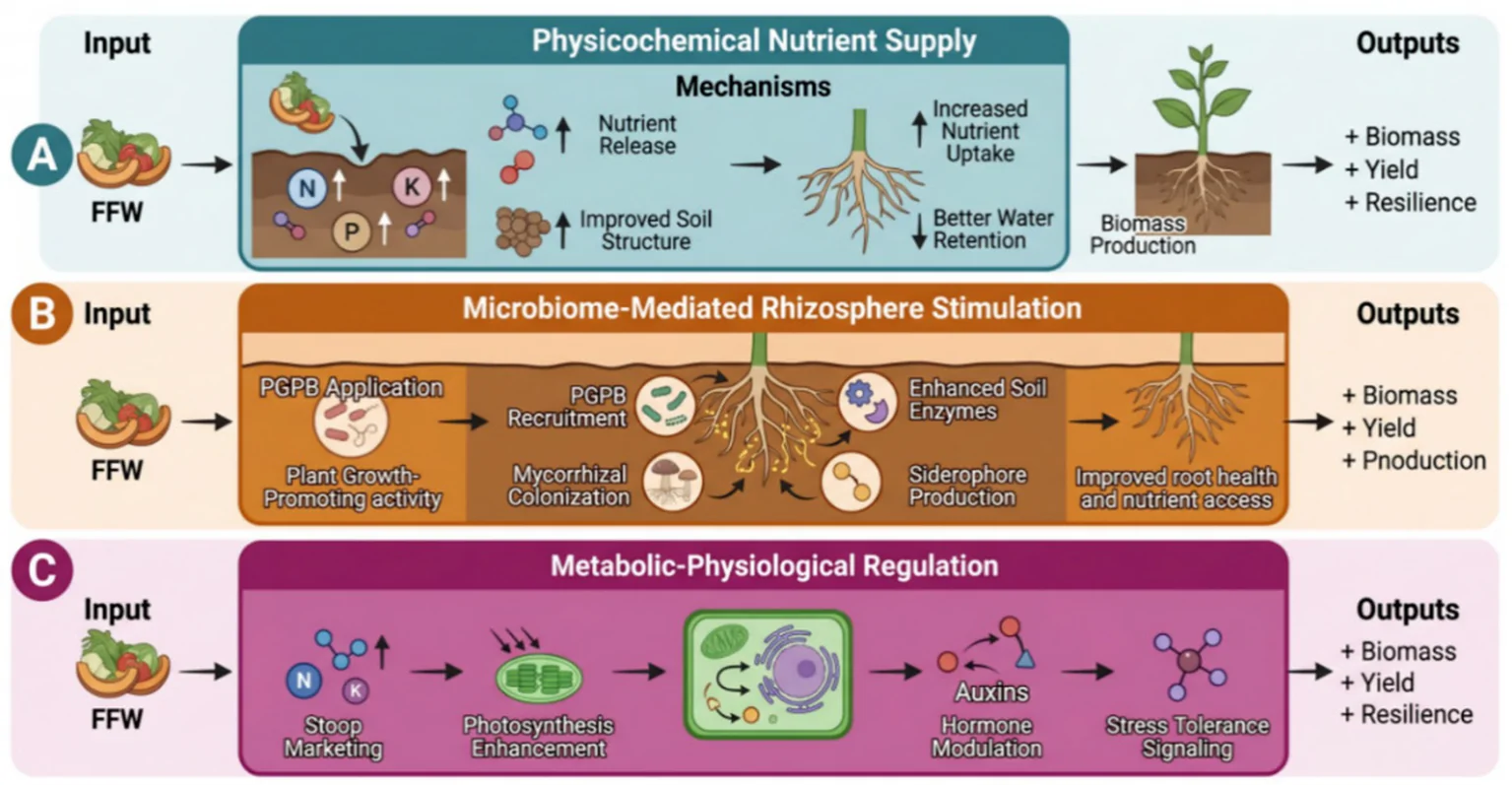
Mechanisms of plant growth regulation by fermented fruit waste (FFW). FFW promotes plant growth through three simultaneous, interacting pathways: **(A)** a physicochemical pathway, in which fermentation-derived organic acids solubilize mineral-bound phosphorus and potassium via chelation and pH modification; **(B)** a microbiome-mediated pathway, in which PGPR-derived signaling molecules, including indole-3-acetic acid (IAA), 1-aminocyclopropane-1-carboxylate (ACC) deaminase, and lipochitooligosaccharide-like signals, modulate root architecture and nutrient transporter expression; and **(C)** a metabolic-physiological pathway, in which fermentation metabolites directly and indirectly influence plant hormonal balance, including auxin and cytokinin signaling, and stimulate antioxidant enzyme expression. These pathways are not independent: nutrient acquisition improves microbial habitat quality at the root surface, reinforcing colonization and creating a positive feedback loop between soil chemistry and microbiome activity that sustains the growth-promoting effect over time.

Nutrient-use efficiency improvements associated with FFW application are also mechanistically linked to microbiome restructuring. By stimulating phosphorus-solubilizing bacteria, nitrogen-mineralizing taxa, and potassium-mobilizing microorganisms whose activity releases plant-available nutrients from mineral-bound and organic pools, FFW effectively synchronizes nutrient delivery with plant demand. Wang et al. (2023) reported that organic amendment application significantly improved N and P use efficiency in wheat cultivation, with concurrent shifts in rhizosphere bacterial community composition toward copiotrophic taxa associated with active nutrient cycling. The three simultaneous pathways through which FFW regulates plant growth, encompassing physicochemical improvement, microbiome-mediated nutrient delivery, and metabolic-physiological modulation, are presented collectively in Figure 6, providing an integrated view of the mechanisms responsible for the consistent agronomic responses documented across crop species and experimental systems.

### 6.2 Enhancement of plant stress resilience

An increasingly prominent application of FFW involves the enhancement of plant tolerance against abiotic stresses including drought, salinity, nutrient deficiency, and oxidative damage, all of which are intensifying under climate-change-driven agricultural conditions. Beneficial rhizosphere microorganisms recruited by FFW application contribute to abiotic stress alleviation through osmolyte accumulation, activation of antioxidant enzyme systems including superoxide dismutase (SOD), catalase (CAT), and peroxidase (POD), and modulation of plant stress-responsive signaling pathways (Vurukonda et al., 2016; Niu et al., 2023). Yang et al. (2022) demonstrated that rhizobacterial communities associated with organic amendment applications significantly upregulated induced systemic resistance (ISR) pathways in wheat under drought stress, reducing oxidative damage markers by 30–45% compared with unamended treatments.

**Figure 7:**
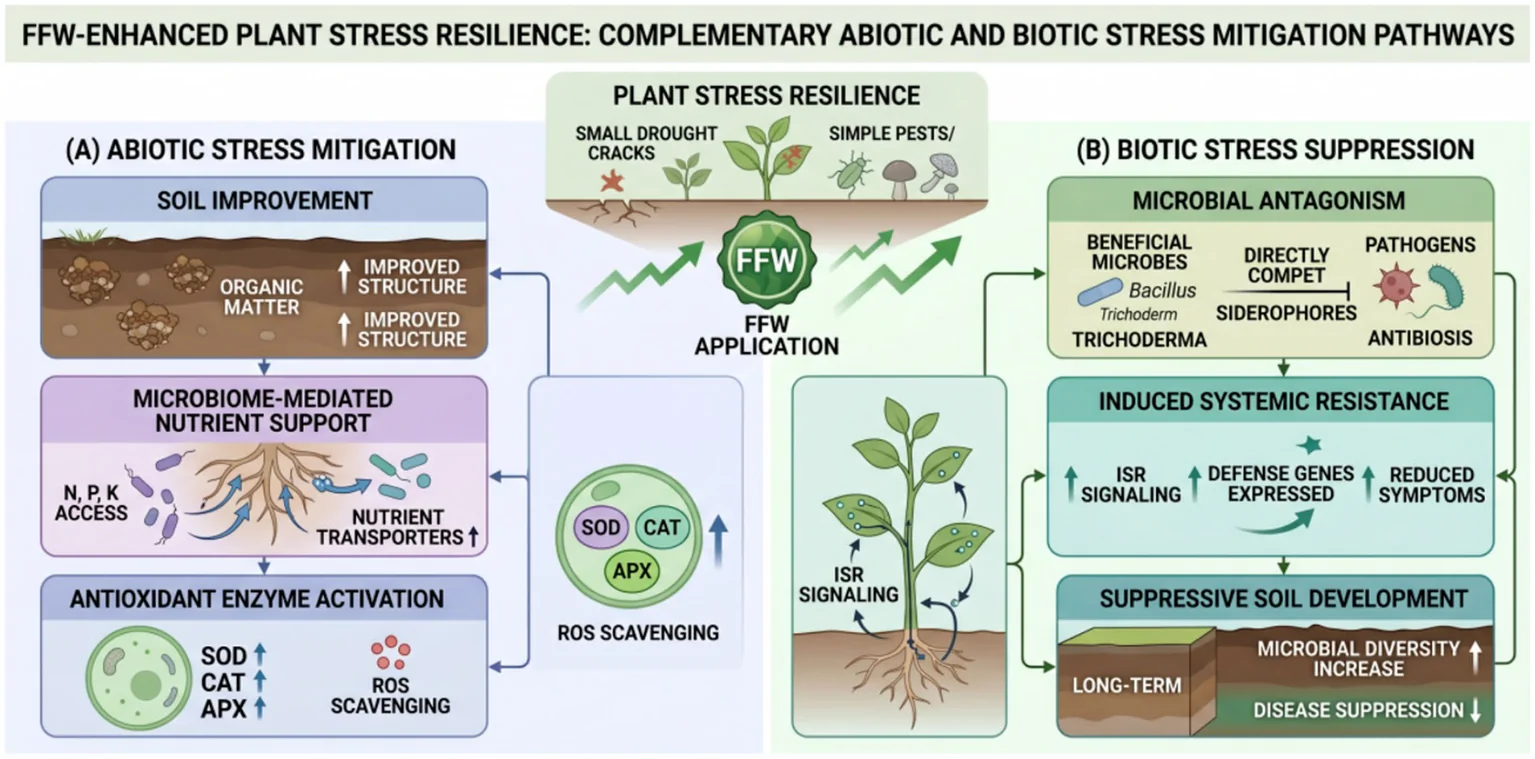
Enhancement of plant stress resilience by FFW. FFW strengthens plant tolerance through two complementary pathways: **(A)** abiotic stress mitigation encompassing soil physical improvement, microbiome-mediated nutrient acquisition, and activation of antioxidant enzyme systems (SOD, CAT, POD); and **(B)** biotic stress suppression through microbial antagonism, induction of systemic resistance (ISR), and suppressive soil development driven by enhanced microbial diversity. FFW, fermented fruit waste; PGPR, plant growth-promoting rhizobacteria; ISR, induced systemic resistance.

Soil organic matter enrichment from FFW application directly supports drought resilience by improving water-holding capacity, reducing evapotranspiration losses, and stabilizing rhizosphere moisture gradients (Blanco-Canqui, 2017). These physical improvements are amplified by microbiome-mediated effects at the root surface, where exopolysaccharide-producing bacteria create hydrated microenvironments that buffer root cells against rapid desiccation. For biotic stress, FFW-enriched microbial communities provide disease suppression through antagonistic activity against soil-borne pathogens, as described in Section 5, with Bacillus-dominated consortia showing particular efficacy against Fusarium, Pythium, and Rhizoctonia species (Ongena and Jacques, 2008; Cha et al., 2016). The two complementary stress-protective pathways through which FFW strengthens plant resilience, covering both abiotic stress mitigation and biotic stress suppression, are illustrated in Figure 7, highlighting the central role of microbiome-mediated mechanisms in reducing oxidative damage, improving water-use efficiency, and establishing suppressive soil conditions that protect crops against pathogen pressure.

### 6.3 Environmental implications of fruit waste and the role of fermentation

The global food system currently generates approximately 1.05 billion tonnes of food waste annually, of which fruit residues constitute a biologically reactive and rapidly decomposing fraction that poses acute environmental management challenges (United Nations Environment Programme, 2024). Disposal of these residues through landfilling generates methane a greenhouse gas with a 20-year global warming potential approximately 80 times greater than carbon dioxide, while composting and natural decomposition in open environments contribute to nitrogen and phosphorus losses, leachate formation, and soil and water quality degradation (Pal et al., 2024; Nguyen et al., 2025). Controlled fermentation redirects this decomposition trajectory, channeling microbial metabolism toward substrate stabilization and nutrient recovery rather than greenhouse gas emission and environmental contamination (Mandal et al., 2024; Sarker et al., 2024).

A quantifiable sustainability advantage of FFW lies in its nutrient circularity potential. Fruit residues carry significant loads of organic carbon, potassium, phosphorus, calcium, magnesium, and trace elements that are largely lost during conventional landfill disposal (Kaushik et al., 2025). Biological recycling through fermentation and subsequent soil application allows these nutrients to re-enter productive agricultural cycles, partially restoring nutrient loops disrupted by intensive farming and reducing external fertilizer demand. Although FFW cannot replace mineral fertilizers in high-input systems on its own, evidence from Li et al. (2020) and Kumar et al. (2025) suggests that regular FFW application can reduce synthetic nitrogen and phosphorus fertilizer requirements by 20–30% in vegetable and cereal production systems through improved soil biological fertility.

### 6.4 Agricultural and environmental benefits of FFW application

Beyond direct nutrient recovery, FFW application may contribute to climate-change mitigation through soil carbon sequestration. Repeated amendment applications that increase soil organic matter content and promote aggregate formation can enhance soil carbon stabilisation, particularly in degraded or carbon-depleted agricultural soils (Lehmann and Kleber, 2015). Khanna et al. (2024) identified fermentation-based organic recycling as a promising strategy within circular bioeconomy frameworks for closing nutrient loops while simultaneously supporting long-term soil carbon storage, a dual-benefit outcome of growing importance in climate-adaptive agriculture. The integration of FFW into decentralized agricultural systems also supports economic resilience and agricultural self-sufficiency, particularly in smallholder and peri-urban farming contexts. Fruit residues are generated continuously across urban and peri-urban environments, creating opportunities for localized fermentation and soil-amendment production that reduce transportation requirements, landfill dependency, and waste-management costs (Kolawole et al., 2025; Garg et al., 2025). In low-input farming systems, low-cost LAB-based fermentation approaches using naturally occurring microbial inocula represent accessible and scalable strategies for improving soil fertility without reliance on imported chemical fertilizers.

### 6.5 Risks, limitations, and quality-control requirements

The agronomic potential of FFW is well documented, but its practical deployment is constrained by a series of ecological, microbiological, and technical risks that must be systematically managed to ensure both environmental safety and consistent performance. These risks arise from the inherent heterogeneity of fruit waste feedstocks, the variability of fermentation processes, and the complexity of soil–amendment–plant interactions that determine real-world outcomes. Table 4 provides a structured overview of the primary risk categories, their causative factors, potential impacts, and evidence-based mitigation strategies.

Phytotoxicity is the most immediate risk associated with immature or improperly stabilized FFW. Organic residues undergoing incomplete fermentation accumulate elevated concentrations of low-molecular-weight organic acids, soluble salts, phenolic compounds, or ammonia that inhibit root elongation, disrupt seed germination, and suppress rhizosphere microbial equilibrium (Bernal et al., 2009; Alburquerque et al., 2012). The germination index calculated from the germination percentage and root length of a standard test species relative to a water control remains the most widely accepted single indicator of amendment phytotoxicity risk, with a threshold value above 50% generally considered indicative of agronomic safety. However, no single physicochemical indicator is sufficient in isolation; comprehensive maturity assessment integrating pH, electrical conductivity, C: N ratio, organic acid profile, and germination testing provides the most reliable quality-control framework (Bernal et al., 2009).

Microbial safety is a further critical concern, particularly for FFW derived from mixed urban or household waste streams. Although properly conducted fermentation at sustained pH below 4.0 effectively suppresses major human pathogens, including Salmonella, *Escherichia coli*, and Listeria species, through organic acid accumulation and competitive exclusion, poorly managed systems may permit the survival of these organisms if acidification is insufficient or inconsistent (Raman et al., 2022). An emerging and underappreciated risk involves antibiotic resistance gene (ARG) transfer: recent microbiome-oriented research by Wang et al. (2023) demonstrated that waste-derived amendments originating from hospital or urban sources may introduce ARGs into agricultural soil ecosystems, with potential long-term consequences for soil microbiome health and food safety.

Contaminant accumulation represents a persistent limitation for FFW derived from non-certified waste streams. Fruit residues from commercial markets and processing facilities may contain pesticide residues, heavy metals, and synthetic additives that can persist through fermentation and accumulate in soil following repeated amendment applications (Sharma K. et al., 2017; Sharma B. et al., 2017; Corradini et al., 2021). Microplastic contamination, a growing concern across all waste-derived amendments, warrants particular attention given its documented effects on soil microbial diversity and invertebrate ecology. Addressing these contamination risks requires feedstock certification, pre-screening protocols, and, where possible, restriction of FFW production to certified organic waste sources.

The absence of standardized fermentation protocols and universally accepted quality indicators remains a major structural limitation of the field. Current studies employ widely varying fermentation conditions, inoculum compositions, substrate ratios, and maturity criteria, making cross-study comparisons difficult and limiting the reproducibility of agronomic outcomes under field conditions (Sarker et al., 2024; De Vuyst and Leroy, 2020). Most existing evidence is also derived from short-term laboratory or pot-scale experiments, with long-term field validation across diverse agroecosystems remaining sparse. Addressing these knowledge gaps requires coordinated efforts to develop internationally accepted fermentation standards, microbiome-based quality indicators, and multi-site field validation networks.

## 7 Conclusions and future perspectives

The evidence synthesized in this review supports a clear repositioning of FFW within the landscape of sustainable agricultural inputs. Fruit waste, when subjected to controlled microbial fermentation, is transformed from an ecologically disruptive and economically burdensome residue into a biologically active amendment capable of simultaneously influencing soil physicochemical properties, microbiome structure, rhizosphere dynamics, plant nutrition, and stress resilience. The consistency of documented outcomes soil organic carbon increases of 12 to 24%, microbial biomass improvements of 38 to 64%, and plant growth enhancements of 15 to 35% across diverse experimental systems provides a quantitative foundation for the agronomic value of FFW that goes beyond qualitative claims.

Three conclusions emerge with particular clarity from this synthesis. First, the beneficial effects of FFW are primarily microbiome-mediated rather than nutrient-driven. The restructuring of soil microbial communities through selective enrichment of PGPR, phosphorus-solubilizing bacteria, and antagonistic taxa represents the mechanistic core of FFW’s agronomic function. Second, fermentation quality is not merely a processing consideration it determines whether FFW functions as a beneficial amendment or a source of phytotoxic, saline, or microbiologically unsafe material. Third, the environmental value of FFW extends beyond nutrient recycling to include greenhouse gas emission reduction, soil carbon sequestration, and the partial substitution of energy-intensive synthetic fertilizers within circular agricultural systems.

Future research should prioritise four needs, each paired with a proposed experimental approach to address it. First, standardized metrics for soil health post-fermentation are needed, which could be achieved through the adoption of a common minimum reporting set, including soil organic carbon, microbial biomass carbon, a defined enzyme panel, and a standardized phytotoxicity/germination index, across future FFW field trials to enable cross-study comparison. Second, long-term field trials across climates remain scarce, and coordinated multi-site trials spanning at least three growing seasons across contrasting climatic zones, following a shared experimental protocol, would help address this gap. Third, life-cycle assessments that jointly evaluate environmental and economic performance are needed, ideally through paired LCA-TEA (techno-economic assessment) studies that assess both dimensions together rather than in isolation. Fourth, regulatory and safety studies remain underdeveloped, and systematic pathogen survival and heavy-metal/contaminant screening across feedstock sources, benchmarked against existing compost safety standards, would help establish FFW-specific safety thresholds. Future field validation would also benefit from a standardized workflow: baseline, mid-season, and end-of-season sampling, a core assay panel (soil organic carbon, microbial biomass carbon, enzyme activity, 16S rRNA sequencing), and consistent unamended and mineral-fertilizer controls. Outcome metrics should likewise be standardized, reporting both total and plant-available nitrogen, microbial biomass carbon, and greenhouse gas fluxes per unit area and time. Pairing multi-omics profiling with greenhouse and field trials of at least two growing seasons would help distinguish transient from persistent effects, while wider publication of negative results and replication studies would reduce publication bias in this field.

From a broader scientific perspective, FFW represents a compelling convergence of fermentation biotechnology, soil ecology, plant physiology, and circular economy principles. Its future development as a microbiome-guided agricultural tool will depend on sustained interdisciplinary collaboration and a commitment to rigorous quality assurance conditions without which neither its agronomic potential nor its environmental promise can be fully realized.
